# Cold adaptation and replicable microbial community development during long-term low-temperature anaerobic digestion treatment of synthetic sewage

**DOI:** 10.1093/femsec/fiy095

**Published:** 2018-05-25

**Authors:** C Keating, D Hughes, T Mahony, D Cysneiros, U Z Ijaz, C J Smith, V O'Flaherty

**Affiliations:** 1 Microbiology, School of Natural Sciences and Ryan Institute, National University of Ireland, Galway, Ireland; 2 Infrastructure and Environment, School of Engineering, University of Glasgow, Rankine Building, 79-85 Oakfield Avenue, Glasgow, G12 8LT, UK

**Keywords:** anaerobic digestion, psychrophilic, hydrolysis, microbial community structure, adaptation

## Abstract

The development and activity of a cold-adapting microbial community was monitored during low-temperature anaerobic digestion (LtAD) treatment of wastewater. Two replicate hybrid anaerobic sludge bed-fixed-film reactors treated a synthetic sewage wastewater at 12°C, at organic loading rates of 0.25–1.0 kg chemical oxygen demand (COD) m^−3^ d^−1^, over 889 days. The inoculum was obtained from a full-scale anaerobic digestion reactor, which was operated at 37°C. Both LtAD reactors readily degraded the influent with COD removal efficiencies regularly exceeding 78% for both the total and soluble COD fractions. The biomass from both reactors was sampled temporally and tested for activity against hydrolytic and methanogenic substrates at 12°C and 37°C. Data indicated that significantly enhanced low-temperature hydrolytic and methanogenic activity developed in both systems. For example, the hydrolysis rate constant (*k*) at 12°C had increased 20–30-fold by comparison to the inoculum by day 500. Substrate affinity also increased for hydrolytic substrates at low temperature. Next generation sequencing demonstrated that a shift in a community structure occurred over the trial, involving a 1-log-fold change in 25 SEQS (OTU-free approach) from the inoculum. Microbial community structure changes and process performance were replicable in the LtAD reactors.

## Teaser

This paper explores low-temperature anaerobic digestion of a synthetic sewage-based wastewater with a focus on microbial community adaptation when using a mesophilic starting community.

## INTRODUCTION

High-rate anaerobic digestion (AD) of domestic wastewaters is both successful and well established at full scale in tropical regions (Bowen *et al.*^2014^). Low-strength anaerobic treatment of wastewater at ambient temperatures in areas with a temperate climate, however, calls for efficient AD processes capable of operating below 20°C. Numerous successful laboratory-scale low-temperature [<20°C] anaerobic digestion (LtAD) trials have been undertaken over the past decade for a range of waste streams (e.g. Connaughton, Collins and O'Flaherty 2006; Enright *et al.*^2009^; McKeown *et al.*^2012^; Gouveia *et al.*^2015^). Yet, despite laboratory-scale success and the economical and environmental advantages of LtAD, full-scale implementation has not yet come to fruition. Moreover, many successful LtAD studies have focused on less complex wastewater and, as such, do not address the issue of solids hydrolysis (Petropoulos *et al.*^2017^). It has been reported that hydrolysis rates decrease as temperatures drop and suspended solids subsequently may accumulate in AD reactors, causing a reduction in treatment efficiencies and biomass washout (Elmitwalli *et al.*^2001^; Singh and Viraraghavan 2002; Lew *et al.*^2003^). Recent studies have, however, demonstrated efficient treatment of sewage by LtAD (Smith, Skerlos and Raskin 2013; Keating *et al.*^2016^) using reactors designed to retain biomass and particulates.

Efficient long-term treatment cannot rely solely on physical entrapment. The degradation of organic matter to methane during LtAD is dependent on the microbial community structure (Raskin *et al.*^1994^) and the activity (Lettinga *et al.*^1999^; Foresti, Zaiat and Vallero 2006; Cavicchioli 2015) of the reactor biomass, which are strongly influenced by temperature. The requirement for a psychrophilic, or psychrotolerant, inoculum for successful LtAD has been proposed as being advantageous. The use of a truly psychrophilic inoculum (from naturally cold environments) has been tested by Xing, Zhao and Zuo (2010) and Petropoulos *et al.* (2017) with promising results. A disadvantage of this approach is that this type of biomass is non-granular and may not have high levels of activity against some substrates. Granular seed inocula are particularly advantageous for biomass settling and retention in high-rate AD reactors (van Lier *et al.*^2001^; Sakar, Yetilmezsoy and Kocak 2009). Granular biomass also provides a more rapid start-up time (Elmitwalli *et al.*^2001^) and can prevent acidification (Neves, Oliveira and Alves 2004). Using mesophilically cultivated granular inocula for psychrophilic treatment without prior efforts to cold-adapt has been deployed in numerous studies, with varying degrees of success (Rebac *et al.*^1995^; Langenhoff and Stuckey 2000; Smith, Skerlos and Raskin 2013). In light of this information, long-term assessments into cold acclimation (how a community adapts to this change in its environment), activity (how active this community will be) and maturation (the sustainability of this adaptation and activity) of cold-adapting AD communities and how these impact treatment efficiencies warrants deeper investigation.

In practice, full-scale treatment facilities still work as a classic ‘black-box’ systems with the microbial community structure and diversity largely unknown. As of yet, there have not been sufficient advances to link what we know about the microbial communities to process optimisation and bio-monitoring of AD a larger scale. Identifying the structure of the microbial community during stable and unstable periods of operation is crucial to understanding treatment parameters, but this in itself is not straightforward. The diversity of the microbial community within an ecosystem is essential for stability, productivity and sustainability (Girvan *et al.*^2005^). This is true for AD reactors, regardless of operating temperature. Levén, Eriksson and Schnürer (2007) reported a higher diversity of species at lower temperatures during operation during mesophilic and thermophilic conditions. Authors have also described a further increase in microbial diversity from mesophilic to psychrophilic conditions (Bialek *et al.*^2012^). The reproducibility of bacterial community structure in reactor systems is debated owing to high functional redundancy and microbial population disparity between reactors and waste streams. It has been reported that changes in microbial community structure in suspended biomass systems can occur, even during stable operation (Fernández *et al.*^1999^), while other authors have reported no changes in a microbial structure, despite perturbation (Akarsubasi *et al.*^2005^). In contrast, other authors Collins, Mahony and O'Flaherty (2006) and Madden *et al.* (2010) found mirroring microbial communities in identical parallel granular reactor set-ups.

The objective of this study was two-fold: (i) to examine the development of microbial structure and activity of a cold-adapting community in replicated parallel LtAD reactors treating a complex, but defined, wastewater; and (ii) to investigate if mirrored microbial community development occurred in the separate LtAD reactors seeded with the same mesophilically cultivated biomass. We hypothesised that a mesophilic granular inoculum would demonstrate cold adaptation as well reproducible reactor performance and reproducible microbial community development in LtAD reactors with stable input and operational parameters.

## MATERIALS AND METHODS

### Reactor design, set-up and operation

This study employed two glass laboratory-scale hybrid sludge bed/fixed-film reactors (R1 and R2) [2.8 l working volume] as described by (Hughes *et al.*^2011^). Both reactors were seeded with 20 g volatile suspended solids (VSS) l^−1^ of anaerobic biomass. Anaerobic sludge granules were obtained from a mesophilic, full-scale, internal circulation reactor, located at the Carbery Milk Products plant in Co. Cork, Ireland. The VSS content of the granules was 119 g VSS l^−1^. The substrate used was synthetic sewage (SYNTHES; Aiyuk and Verstraete 2004) at 500 mg l^−1^ COD_Tot_. The reactors were operated at 12°C in a trial lasting for 889 days. The trial was divided into five phases, each representative of a different hydraulic retention time (HRT) and organic loading rate (OLR; Table 1). The filter unit was replaced on Day 434.

**Table 1.** Reactor operation phases and associated operational conditions.

Phase	1	2	3	4	5
Days	0–104	105–259	260–559	560–665	666–889
HRT^a^	48	36	24	18	12
TEMP^b^	12	12	12	12	12
OLR^c^	0.25	0.33	0.5	0.63	1
VLR^d^	0.5	0.67	1	1.33	2
SLR^e^	0.03	0.03	0.05	0.06	0.1
SLR^f^	0.01	0.02	0.03	0.03	0.05
UV^g^	2.5	2.5	2.5	2.5	2.5

a Temperature (°C).

b Hydraulic retention time (h).

c Organic loading rate (kg COD m^−3^ d^−1^*.

d Volumetric loading rate (m^3^ Wastewater m^−3^ Reactor d^−1^).

e Sludge loading rate (kg COD kg[VSS]^−1^ d^−1^)*.

f Sludge loading rate (m^3^Wastewater kg[VSS]^−1^ d^−1^).

g Up-flow velocity (m h^−1^). *Values calculated based on influent concentration of 500 mg l^−1^ COD_Tot_.

### Reactor effluent analyses

Reactor effluent was sampled daily and also combined into a weekly composite sample for total, chemical oxygen demand (COD) (COD_Tot_), soluble COD (COD_Sol_), suspended COD (COD_Sus_) and colloidal COD (COD_Col_) determinations according to Standard Methods (APHA 2005). Protein and polysaccharide concentrations in the effluent were determined by the Lowry method (Lowry *et al.*^1951^) and the DuBois method (DuBois *et al.*^1956^), respectively. The concentration of volatile fatty acids (VFAs) in the effluent was determined by chromatographic analysis in a Varian Saturn 2000 GC/MS system (Varian Inc., Walnut Creek, CA). Biogas analysis was performed by gas chromatography (Varian Inc., Walnut Creek, CA) according to Standard Methods (APHA 2005)

### Biomass characterisation

#### Specific methanogenic activity testing

To evaluate changes in the hydrolytic and methanogenic capabilities of the seed (Day 0) and reactor biomass (sampled on days 105, 260, 666 and 889), samples were screened using the specific methanogenic activity (SMA) testing method using the pressure transducer technique, as described previously (Colleran *et al.*^1992^; Keating *et al.*^2016^).

#### Substrate (Protein) degradation assays to assess substrate depletion curve for the determination of *K*, *A*_max_ and *K*_m_

The maximum specific activity (*A*_max_), the maximum initial velocity (*Vmax*), the apparent half-saturation constant (*K*_m_) and the first-order hydrolysis constant (*k*) of the seed inoculum and reactor biomass were evaluated on a protein source (solubilised skimmed milk powder). These rates were determined using substrate depletion assays, which were set up similarly to the SMA test described above and the kinetic parameters calculated as described by Bialek, Cysneiros and O'Flaherty (2013). Tests were performed, in triplicate; at 12°C and 37°C using biomass and protein concentrations of 2 g VSS l^−1^ and 2 g COD vial^−1^, respectively.

### DNA/RNA co-extraction from biomass

Genomic DNA and RNA was extracted from granular biomass samples taken from R1 and R2 on Days 0 (I), Days 105 (P1), 236 (P2a), 296 (P2b), 392 (P3a), 531 (P3b), 546 (P3c), 666 (P4) and at the end of the trial (Day 889). Biomass was sampled from the fixed-film filter at two points:—mid-trial (Day 454) and at the end of the trial (Day 889). The nucleic acids were co-extracted by a modification of a phenol extraction method and processed as outlined by Keating *et al.* (2016).

### Quantitative-polymerase chain reaction

Quantitative polymerase chain reaction (qPCR) was carried out for Archaeal and Bacterial domains using DNA and cDNA generated from granular biomass sampled from R1 and R2 and the fixed-film filter as described above. qPCR was performed using a LightCycler 480 (Roche, Manheim, Germany). The primers 1369F and 1492R and *Taq*man probe TM1389F (5′-CTTGTACACACCGCCCGTA-3′) were used for bacterial analysis (Suzuki, Taylor and DeLong 2000). The primers 787F and 1059R and *Taqman* probe 915F (5′-AGGAATTGGC-GGGGGAGCAC-3′) were used for archaeal analysis (Yu *et al.*^2005^). Standard curves were prepared using plasmids containing the full-length 16S rRNA gene sequence from a representative bacterial (*Escherichia coli*) and archaeal (*Methanosarcina bakeri*) strain. The plasmids were extracted using a Plasmid Extraction kit (BIOLINE). A PCR reaction was then carried out using the primer pairs described above. This product was cleaned using QIAQuick PCR Clean Up kit (Qiagen, Crawley, UK) according to manufacturer's instructions. To construct the RT-PCR cDNA standard curves were produced from cDNA prior in vitro transcription of the target mRNA by using the MEGAshortscript T7 kit (Ambion) as described by Smith *et al.* (2006). The concentration of standards was measured in duplicate using a Qubit system (Invitrogen) and converted into copy concentration. A 10-fold serial dilution series (10^9^–10^1^ copies ml^−1^) was generated for each standard solution and analysed, in duplicate, with its corresponding primer and probe set. qPCR cycling conditions can be found in Keating *et al.* (2016).

### Illumina Miseq analysis

Terminal Restriction Fragment Length Polymorphism was used as a screening step to select samples for next generation sequencing (data not shown)-outlined in Keating *et al.* (2016). Subsequently, DNA and cDNA from reactor biomass sampled on Day 0 (Seed), Days 296 (P2b), 531 (P3b), Take-Down (E-Day 889) and from the filter upon take-down (FE) were sent for Miseq Illumina analysis at MR DNA (Shallowater, Texas, USA). Universal 16S rRNA primer pair targeting the V4 region were used, 515F (5′-GTGCCAGCMGCCGCGGTAA-3′) and 806R (5′GGACTACHVGGGTWTCT-AAT-3′)—for paired-end sequencing with the forward primer in each pair containing a barcode sequence. Amplicons were pooled and purified using calibrated Ampure XP beads (Bechman Coulter). This product was prepared using the Illumina TruSeq DNA library protocol. The DNA library was processed on a Solexa Miseq machine according to the manufacturer's instructions. Sequences were analysed using an OTU-free approach using the DADA2 algorithm (Callahan *et al.*^2016^). We used the standard workflow given at http://benjjneb.github.io/dada2/tutorial.html that learns the error model from the data first, dereplicates the reads and then runs the DADA2 algorithm separately on forward and reverse reads. Finally, merging the overlapping reads from both forward reduced sequence variants and reverse reads to give 1396 unique sequences (SEQs), which were then used to create sequence tables for the different samples. The representative SEQs were then taxonomically classified against the Silva 123 database with assign_taxonomy.py script from Qiime (Caporaso *et al.*^2010^). To find the phylogenetic distances between SEQs, we multisequence aligned the SEQs against each other using mafft v7.040 (Katoh and Standley 2013) and FastTree v2.1.7 (Price, Dehal and Arkin 2010). Finally, the make_otu_table.py from Qiime was employed to combine abundance table with taxonomy information. Raw sequences were submitted to the SRA database under bioproject submission number SUB3108010.

### Statistical analysis

GraphPad Prism software (San Diego, California, USA) was used for calculating Student's *t*-test based on reactor effluent parameters and qPCR data. A significance level of 95% (*P* < 0.05) was selected. Further statistical analyses of the sequencing data were performed via the software R, version 3.4.1 (http://www.R-project.org/) using the SEQS tables and data generated as described previously and metadata. For community analysis, we used the package ‘Vegan’ (Oksanen *et al.*^2013^). The following alpha diversity measures were used: Fisher's alpha; Pielou's evenness; Richness; Shannon and Simpson. We used Vegan's aov() to calculate pair-wise ANOVA *P*-values and drew these on top of alpha diversity figures. To calculate Unifrac distances, we used the package ‘Phyloseq’ (McMurdie and Holmes 2013). Principal co-ordinate analysis (PCoA) plot of community data (SEQs) were made using different distance measures (Vegan's capscale() function): Bray Curtis; Unweighted Unifrac; and Weighted Unifrac. The samples were grouped for different treatments as well as the mean ordination value and spread of points (ellipses were drawn using Vegan's ordiellipse() function that represent the 95% confidence interval of the standard errors). To find SEQs that are significantly different between different conditions, we used DESeqDataSetFromMatrix() function from DESeq2 (Love, Huber and Anders 2014) package with adjusted *P*-value (after accounting for all comparisons) cut-off of 0.01 and minimum log fold change of 1. After performing multiple testing corrections, it reports SEQs that have log-fold changes between multiple conditions. The statistical workflows for the above can be found at http://userweb.eng.gla.ac.uk/umer.ijaz#bioinformatics.

## RESULTS

### Reactor performance

Both reactors treated the synthetic sewage wastewater successfully, with COD removal efficiencies in excess of 80% generally recorded, corresponding to low effluent COD concentrations of typically less than 120 mg COD_Tot_ l^−1^ at applied OLRs up to 0.63 kg COD_Tot_ m^−3^ day^−1^ (Table 2). The performance was sustained during the long-term trial, with minor fluctuations, until the loading rate was increased to 1.0 kg COD_Tot_ m^−3^ day^−1^ from Day 666 (Table 1), at which point the efficiency of the process decreased somewhat in R1, although COD_Tot_ removal rates of c. 60% were maintained (Table 2).

**Table 2.** Average effluent COD_Tot_, COD_Sus_, COD_Col_ and COD_Sol_ values during the five phases of reactor operation for R1 and R2. a; concentration in mg l^−1^, b; removal efficiency percentage, c; standard deviation, d; VFA:COD ratio based on average VFA concentrations and COD_Sol_ for each phase.

Sample	Total COD			Suspended COD			Colloidal COD			Soluble COD		
	(Conc)^a^	(RE)^b^	(SD)^c^	(Conc)	(RE)	(SD)	(Conc)	(RE)	(SD)	(Conc)	(RE)	(SD)
R1 Phase 1	73	86	±11	22	71	±8	11	60	±0.1	41	84	±2
R2 Phase 1	140	73	±4	62	17	±0.2	23	17	±5	56	78	±2
R1 Phase 2	61	88	±4	18	75	±6	4	84	±2	40	83	±1
R2 Phase 2	76	85	±6	31	58	±0.7	13	27	±0.7	32	86	±4
R1 Phase 3	110	79	±3	41	46	±6	25	9	±2	45	82	±1
R2 Phase 3	103	80	±19	39	47	±15	24	14	±4	40	84	±2
R1 Phase 4	124	75	±25	46	36	±13	39	0	±6	44	84	±3
R2 Phase 4	105	79	±8	27	63	±14	33	0	±5	46	83	±0.2
R1 Phase 5	215	59	±12	125	0	±0.5	25	9	±1	67	73	±2
R2 Phase 5	114	78	±5	61	19	±0.9	15	45	±1	37	85	±3
VFA:COD (Ratio)^d^	Phase 1	Phase 2	Phase 3	Phase 4	Phase 5							
R1	0.07	0.1	0.14	–	1.47							
R2	0.04	0.14	0.48	–	0.56							

### Replicability of reactor performance

During Phase 1, a significant difference (*P* < 0.05) in performance was observed between the two reactors. Reactor 2 (R2) average COD concentrations were much higher than reactor 1 (R1) for all COD fractions (Table 2). However, this can be attributed to a start-up period of ∼56 days for R2, while no start-up period was observed for R1. Both systems performed well upon commencement of the second phase. Transient increases in the effluent concentrations of the COD_Tot_, COD_Sus_ and COD_Col_ fractions from both reactors were observed upon further reduction of the applied HRT during Phase 3 (Table 2). The COD_Sol_ removal, however, was not noticeably affected by this change (Table 2).

The particulate proportion of the influent (COD_Sus_) was degraded/retained in both reactors until concentrations in effluent from R1 increased from Day 329 (Phase 3) and subsequently effluent COD_Col_ concentrations also increased. This suggested that suspended solids might have been degraded to colloidal particles. Similarly, effluent COD_Sus_ and COD_Col_ in R2 increased during this period. These fractions of COD remained elevated in effluent from both reactors, until the filter matrix was changed on Day 434 (Phase 4). The fourth period of reactor operation was characterised by efficient and stable process performance by both systems, with removal efficiencies of COD_Tot_ and COD_Sol_ routinely >75% (Table 2). However, colloidal particles were not degraded/retained by either R1 or R2 (0% removal). The removal of the COD_Tot_, COD_Sol_ and COD_Sus_ fractions was not significantly different (*P* > 0.05) between the replicate reactors during phases 2–4.

The final operational phase (Phase 5) was defined by an HRT of 12 h and an applied OLR of 1 kg COD m^−3^ d^−1^. The response to this HRT change perturbation was distinct in both reactors. A period of biomass washout, lasting two weeks, upon commencement of the phase was observed in R1, with effluent COD_Tot_ concentrations reaching 1 g l^−1^, composed primarily of suspended solids, before slowly decreasing over a period of 20–25 days. In contrast, R2 displayed no obvious response to the HRT change. The effluent VFA to COD ratio was highest during this phase (Table 2). A period of ∼100 days of stable operation was then recorded in both reactors before R1 effluent values began to fluctuate again, with COD_Sus_ concentrations reaching 440 mg l^−1^ on Day 868. R2 also displayed a period of less efficient performance from Day 819, where COD_Sus_ and COD_Sol_ increased (reaching below 130, and 150 mg l^−1^, respectively). An increase in effluent VFA concentrations was recorded from Day 805, to reach a range of 10–20 mg l^−1^ (data not shown). It was demonstrated that effluent COD_Tot_ and COD_Sol_ were significantly different (*P* < 0.05) between both systems during this final phase of the trial.

Protein was completely hydrolysed/degraded in both reactors throughout the trial with removal efficiencies of c. 100% (Table 3). Carbohydrate (the polysaccharide portion) was also completely degraded/retained in both reactors (Table 3) until the filter matrix was changed on Day 434. Following this, effluent carbohydrate concentrations from R1 reached 34 mg l^−1^ on Day 490 (data not shown). The removal of carbohydrates was not significantly different between systems during phases 1–4, but during the final phase of operation *P* was < 0.05.

**Table 3.** Average effluent Carbohydrate and Protein values throughout the five phases of reactor operation for R1 and R2. a; concentration in mg l^−1^, b; removal efficiency percentage, c; standard deviation.

Sample	Carbohydrate			Protein		
	(Conc)^a^	(RE)^b^	(SD)^c^	(Conc)	(RE)	(SD)
R1 Phase 1	0	100	±0	0.05	100	±0.04
R2 Phase 1	0	100	±0	0.01	100	±0.05
R1 Phase 2	0.3	100	±1	0.08	99	±0.05
R2 Phase 2	0.1	100	±0.05	0.03	100	±0.1
R1 Phase 3	7.08	91	±1	0.09	99	±0.04
R2 Phase 3	4.1	95	±1	0.07	99	±1
R1 Phase 4	12	84	±1	0	100	±0
R2 Phase 4	24.3	69	±3	0.02	100	±0.02
R1 Phase 5	9.4	88	±8	0.01	100	±0.02
R2 Phase 5	2.1	98	±1	0	100	±0.06

### Microbial activity and cold adaptation

The granular biomass was sampled temporally from each reactor throughout the trial and tested for its activity against hydrolytic and methanogenic substrates under mesophilic and psychrophilic conditions to; assess: (i) the activity of the microbial population; (ii) if the microbial populations were adapting to psychrophilic conditions; and (iii) if the activity and adaptation developed at the same rate in both reactor systems.

#### Hydrolysis

The hydrolysis rate constant (*k*) results demonstrated that, throughout the trial, biomass activity increased when tests were carried out under both mesophilic and psychrophilic conditions. In fact, the hydrolysis rate at 12°C during phase 4 was increased by ∼20 times in biomass from both reactors, compared to the seed inoculum. In biomass from both reactors, A_max_ (Table 4) increased at both temperatures tested, with psychrophilic activity at the end of the trial being comparable to (R1), or greater than (R2) the mesophilic activity. K*_m_* for mesophilic hydrolysis increased throughout the trial for both reactors, indicating a decrease in substrate affinity at the higher temperature. K*_m_* at the lower temperature decreased over time, indicating an increase in substrate affinity for both biomass sources under these conditions.

**Table 4.** Hydrolysis kinetic assays of reactor biomass at 37°C and 12°C, based on a skimmed milk protein source. a; Maximum substrate utilising rate gCOD gProtein^−1^ d^−1^. b; Apparent half-saturation rate constant gProtein l^−1^. c; Maximum initial velocity gProtein l^−1^ d^−1^ for R1 and R2, d: Hydrolysis rate constant d^−1^. Values are the mean of triplicates ± standard deviation in brackets.

Sample	A*_max_*^a^	K*_m_*^b^	*Vmax* ^c^	*k^d^*
Seed 37°C	15 (±3)	1.1 (±0.41)	0.9 (±1.07)	0.9 (±0.58)
Seed 12°C	18 (±0.13)	2.5 (±0.03)	0.3 (±0.05)	0.3 (±0.06)
R1 Phase 1 37°C	12 (±1)	1.8 (±0.36)	2.2 (±1.13)	1 (±0.42)
R2 Phase 1 37°C	104 (±10)	2.7 (±0.07)	0.8 (±0.23)	0.8 (±0.31)
R1 Phase 1 12°C	40 (±7)	1 (±0.34)	1.1 (±0.4)	1.3 (±0.06)
R2 Phase 1 12°C	19 (±2.52)	4.3 (±3)	2.7 (±2.19)	0.9 (±0.21)
R1 Phase 2 37°C	145 (±26.5)	3.9 (±2.12)	8.8 (±8.39)	5.7 (±2.18)
R2 Phase 2 37°C	164 (±16)	3.1 (±2.23)	7.5 (±6.72)	2.1 (±0.91)
R1 Phase 2 12°C	127.5 (±45)	1.6 (±0.11)	0.1 (±0.04)	0.8 (±0.16)
R2 Phase 2 12°C	35 (±27)	1.4 (±0.13)	0.1 (±0.06)	1.2 (±0.58)
R1 Phase 3 37°C	94 (±26)	1.6 (±0.43)	3.3 (±3.94)	1.6 (±0.37)
R2 Phase 3 37°C	62 (±9.5)	2.8 (±0.14)	3.1 (±0.70)	2.2 (±0.41)
R1 Phase 3 12°C	52 (±32)	2.6 (±0.19)	0.3 (±0.09)	1.1 (±0.32)
R2 Phase 3 12°C	91 (±29)	1.9 (±0.22)	0.3 (±0.16)	1.5 (±0.33)
R1 Phase 4 37°C	257 (±32)	1.8 (±0.27)	1 (±1.07)	0.9 (±0.58)
R2 Phase 4 37°C	15 (±1)	2.1 (±2.02)	2 (±2)	1.9 (±1.18)
R1 Phase 4 12°C	40 (±18)	0.5 (±0.04)	0.1 (±0.01)	3.2 (±0.89)
R2 Phase 4 12°C	51 (±5)	0.3 (±0.08)	1.7 (±1.57)	4.7 (±2.92)
R1 End 37°C	89 (±33)	2.8 (±1.58)	2.3 (±3.73)	1.4 (±0.787)
R2 End 37°C	72 (±8)	1.2 (±0.16)	1 (±0.27)	2.9 (±0.216)
R1 End 12°C	68 (±7)	1.8 (±0.37)	0.4 (±0.23)	1.8 (±0.714)
R2 End 12°C	83 (±22)	2.2 (±0.19)	0.6 (±0.15)	1.6 (±0.383)

#### Acetogenic and direct methanogenic substrates

The initial inoculum had a high SMA at 37°C, with acetoclastic activity being six times higher than hydrogenotrophic activity (Table 5). While SMA against acetate was only slight (7±1 ml Methane (CH_4_) g [VSS]^−1^ d^−1^) at 12°C, hydrogenotrophic activity (39±19 ml Methane (CH_4_) g [VSS]^−1^ d^−1^) was comparable to that measured at 37°C (Table 5). By the end of the first phase of the trial, reactor biomass SMA at 37°C had increased, with the hydrogenotrophic activity having increased to a level 10 times and 3 times greater than the seed biomass in R1 and R2, respectively. At the lower temperature, there was little change in SMA values compared to the inoculum.

**Table 5.** Maximum specific methanogenic activity (SMA) of reactor biomass at 37°C and 12°C presented as ml Methane (CH_4_) g [VSS]^−1^ d^−1^ for R1 and R2. Values are the mean of triplicates ± standard deviation in brackets.

Sample	Propionate	Butyrate	Ethanol	Acetate	H_2_/CO_2_
Seed 37°C	84 (±9)	523 (±30)	561 (±140)	300 (±33)	50 (±5)
Seed 12°C	5 (±2)	3 (±2)	7 (±4)	7 (±1)	39 (±19)
R1 Phase 1 37°C	100 (±26)	155 (±19)	470 (±39)	345 (±20)	570 (±115)
R2 Phase 1 37°C	30 (±3)	55 (±18)	166 (±45)	179 (±32)	134 (±6)
R1 Phase 1 12°C	8 (±3)	2 (±15)	33 (±13)	14 (±11)	15 (±2)
R2 Phase 1 12°C	5 (±3)	6 (±1)	51 (±18)	14 (±7)	31 (±3)
R1 Phase 2 37°C	136 (±20)	147 (±35)	230 (±97)	79 (±47)	65 (±41)
R2 Phase 2 37°C	87 (±4)	187 (±10)	307 (±46)	11 (±6)	193 (±41)
R1 Phase 2 12°C	17 (±8)	27 (±21)	39 (±2)	0	11 (±3)
R2 Phase 2 12°C	22 (±2)	2 (±2)	12 (±3)	0	24 (±1)
R1 Phase 4 37°C	23 (±2)	65 (±9)	257 (±92)	201 (±18)	170 (±15)
R2 Phase 4 37°C	40 (±22)	94 (±7)	21 (±9)	353 (±50)	308 (±21)
R1 Phase 4 12°C	1 (±1)	3 (±1)	31 (±6)	51 (±22)	19 (±9)
R2 Phase 4 12°C	3 (±1)	12 (±8)	46 (±12)	42 (±6)	98 (±23)
R1 End 37°C	23 (±13)	91 (±13)	153 (±33)	329 (±66)	231 (±29)
R2 End 37°C	34 (±2)	73 (±14)	171 (±16)	181 (±29)	110 (±6)
R1 End 12°C	2 (±1)	9 (±1)	21 (±6)	26 (±7)	38 (±3)
R2 End 12°C	4 (±2)	8 (±3)	29 (±4)	28 (±3)	35 (±4)

R2 biomass sampled during the second phase had an increased SMA against all substrates tested at 37°C, with the exception of acetate. In contrast, R1 biomass displayed decreased activity for all substrates tested, except for propionate. Biomass from both systems tested at the psychrophilic condition demonstrated similar activity ranges with increased activity noted against propionate (Table 5). Interestingly, no acetoclastic activity was detected in either reactor biomass at this point.

At the end of the fourth phase, the SMA of biomass from both systems was within a similar range. Under psychrophilic conditions, the SMA profile had increased for both R1 and R2, with comparable levels of activity in both biomass sources (Table 5). At the end of trial, the SMA at 37°C was comparable for R1 and R2 biomass samples, with the exception of the direct methanogenic substrates, for which SMA was significantly lower in R2 biomass. The SMA at 12°C against all substrates was in a similar range for both R1 and R2 and compared to the initial inoculum activity on ethanol and acetate had increased by ∼3 times (Table 5).

### Microbial community structure

**Figure 1.**
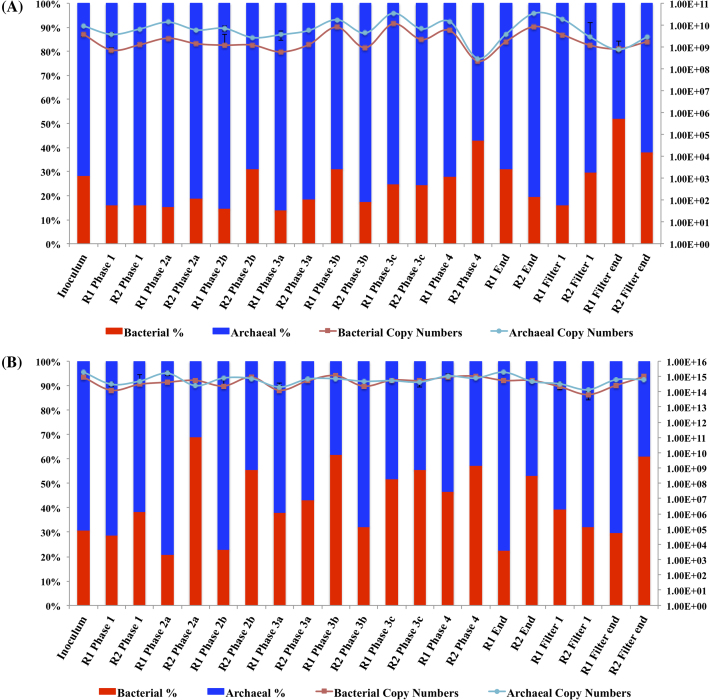
(**A**) qPCR data of Bacterial and Archaeal 16S copy numbers (per g biomass) on the right y-axis from biomass samples (x-axis) throughout the trial corresponding to the ratio of bacteria to archaea (expressed as a percentage) on the left y-axis. (**B**) qPCR data of Bacterial and Archaeal 16S rRNA transcripts copy numbers (per g biomass) on the right y-axis from biomass samples (x-axis) throughout the trial corresponding to the ratio of bacteria to archaea (expressed as a percentage) on the left y-axis.

Bacterial and archaeal numbers were quantified throughout the reactor trial. The bacterial and archaeal profiles were generally reproducible in both systems with copy numbers in the range of 3 × 10^8^–3.5 × 10^10^ (copies g-^1^) and 2.4 × 10^8^–1.2 × 10^10^ (copies g-^1^), respectively (Fig. 1A). The ratio of bacteria to archaea in R1 and R2 biomass was broadly similar also, but deviations were noted, for example, in R2 on Day 296 (Phase 2b) and Day 666 (Phase 4-start of Phase 5), whereby the bacterial population increased to 31% and 43% of the total population, respectively (1.2 × 10^9^ and 2.3 × 10^8^ copies g-^1^) and was greater than in R1 biomass during these times. However, no significant difference (*P* > 0.05) was found throughout the trial. A 1-log reduction of the total bacterial and archaeal gene copy numbers was also observed in R2 biomass on Day 666 (Phase 4-start of Phase 5; Fig. 1A). The filter communities were distinct from the other sampling points with a greater proportion of bacterial to archaeal cells. This was due to a reduction in the numbers of archaeal cells relative to the granular biomass (Fig. 1A).

**Figure 2.**
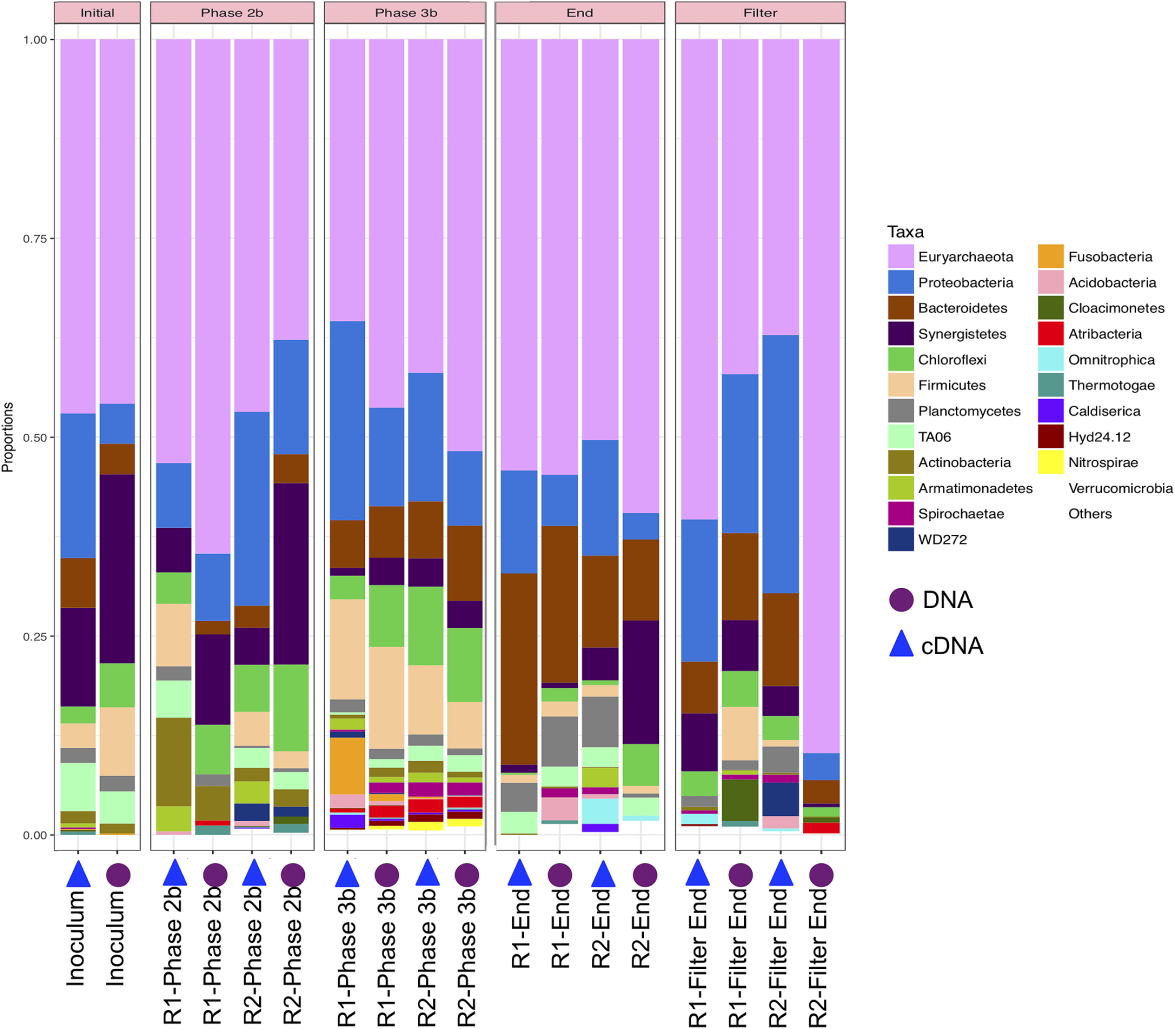
Taxa-plot of the percentage abundance of bacterial and archaeal phyla identified per sample. Samples are grouped according to phase ‘Initial’, ‘Phase 2’, ‘Phase 3’, ‘End’ and ‘Filter’.

The 16S rRNA transcripts (Fig. 1B) varied from 6.5 × 10^13^ to 1.15 × 10^15^ copies g−^1^ (21%–69%) for bacterial cells and 1.4 × 10^14^ to 2 × 10^15^ copies g−^1^ (31%–79%) for archaeal cells, these numbers were greater than those observed through DNA-based analysis. Deviations were again noted in the proportion of bacteria to archaea between the systems in biomass sampled from Day 236 (Phase 2a), Day 296 (Phase 2b), Day 531 (Phase 3b), Day 666 (Phase 4-start of Phase 5), End and Filter End (Day 889). The greatest deviation was noted in the biomass sampled on Day 531 (Phase 3b) in which R1 had the highest bacterial transcripts recorded (1.2 × 10^15^ copies g−^1^), comprising 62% of the total sample pool (Fig. 1B). This contrasted with the same time point in R2, when bacterial copy numbers were 2.3 × 10^14^ (copies g−^1^), comprising just 32% of the total sample pool (Fig. 1A). Despite these deviations, the bacterial and archaeal transcript numbers were reproducible between the systems and no significant difference was found between the systems (*P* > 0.05). Nextgeneration sequencing was carried out to identify the bacterial and archaeal populations. The major bacterial populations identified included representatives of the Proteobacteria (8%–52%), mainly Deltaproteobacteria based on a DNA-based analysis and Gammaproteobacteria, based on the cDNA-derived sequences (Fig. 2). The Synergistetes (1.5%–44%) and the Bacteroidetes, mainly Flavobacteria, Sphingobacteria and Bacteroidea (0%–52%) were also present in the reactors throughout the trial. Chloroflexi (0%–19%), Firmicutes comprised mainly Clostridia and Bacilli (0%–24%). Less abundant, or occasionally present, bacterial groups included the Fusobacteria (0%–11%), the Actinobacteria (Actinobacteria and Coriobacteria; 0%–24%), the Planctomycetes (Phycisphaerae and Planctomycetales; 0%–14%), Acidobacteria (Halophagae; 0%–6.5%), and <5% abundance; Caldiserica, Chlorobi, Gemmatimonadetes, Hyd24–12, Omnitrophica, Spirochaetae, Thermotogae, TM6, WD272, Verrucomicrobia and rare phyla; Candidate division SR1, Cyanobacteria, Deferribacteres, Dictyoglomi, Elusimicrobia, Gracilibacteria, Fibrobacteres, Hydrogenedentes, Lentisphaerae, Nitrospirae, Parcubacteria, SHA-109.

**Figure 3.**
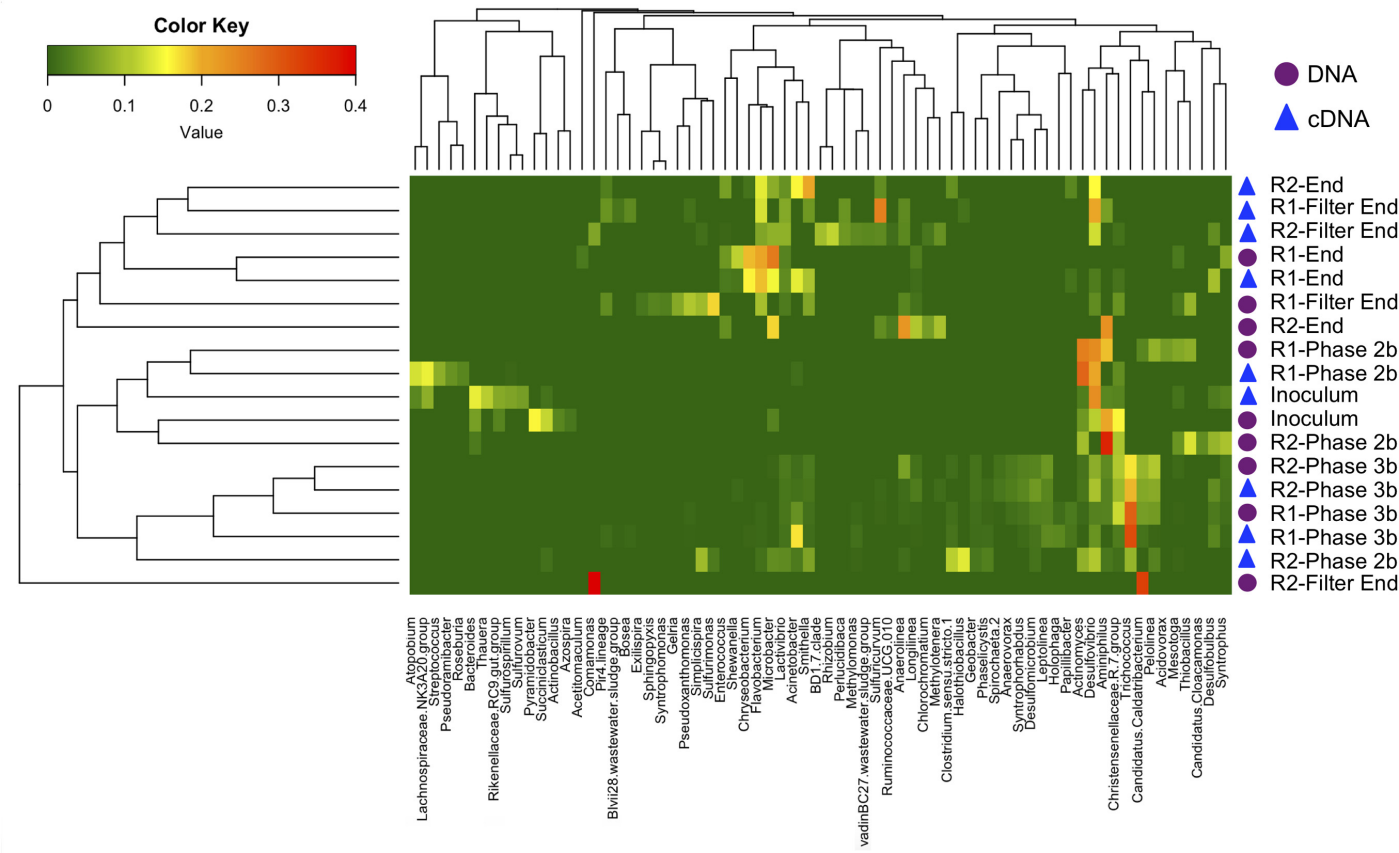
Heatmap analysis for the bacterial throughout the trial showing the dominant genera (>2%) and Bray–Curtis similarity between samples and between the dominant genera.

Heatmap analysis was employed to visualise temporal variations in the bacterial populations in both reactors and similarity matrices were used in tandem (Fig. 3). In the heatmap of the bacterial genera, the sequences clustered together based on time, and DNA or cDNA origin. An exception to this was the R2 DNA biomass sample from the pumice filter unit, which formed a separate branch. This was due to the apparent increased abundance presence of *Commamonas* and *Candidatus Caldatribacterium* and this sample also demonstrated decreased species richness.

**Figure 4.**
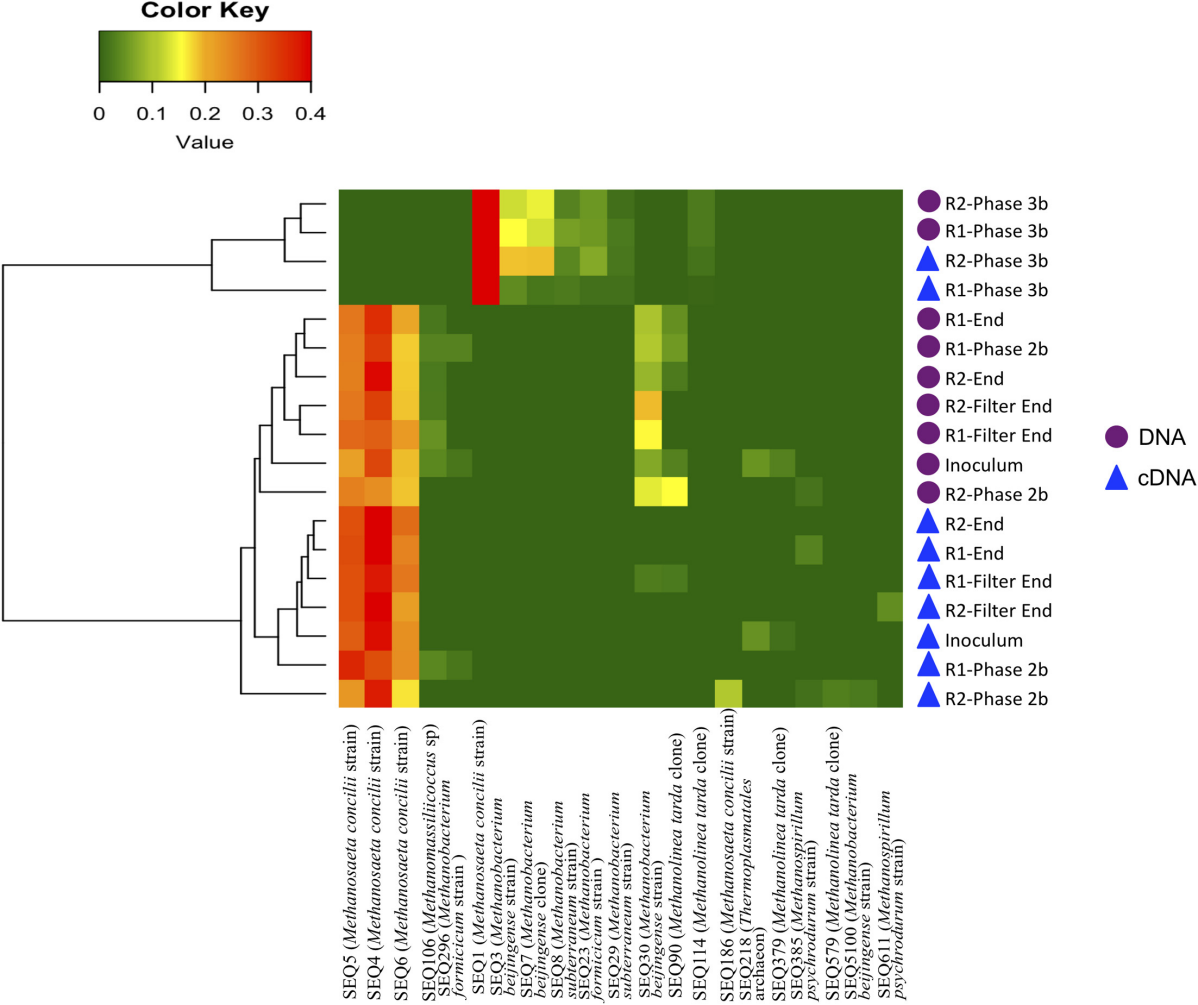
Heatmap analysis for the archaeal fraction throughout the trial showing the dominant sequences (>2%) and Bray–Curtis similarity between samples.

The archaeal portion of the community was dominated by sequences identified as *Methanosaeta concilii* strain X16932 throughout the trial (Fig. 4). Methanobacterium, Methanolinea and Methanospirillum sequences were also present. Biomass from ‘Phase 3b’ branched separately due to apparent decreases in hydrogenotrophic methanogens.

### Microbial community development over time

#### Community comparisons

**Figure 5.**
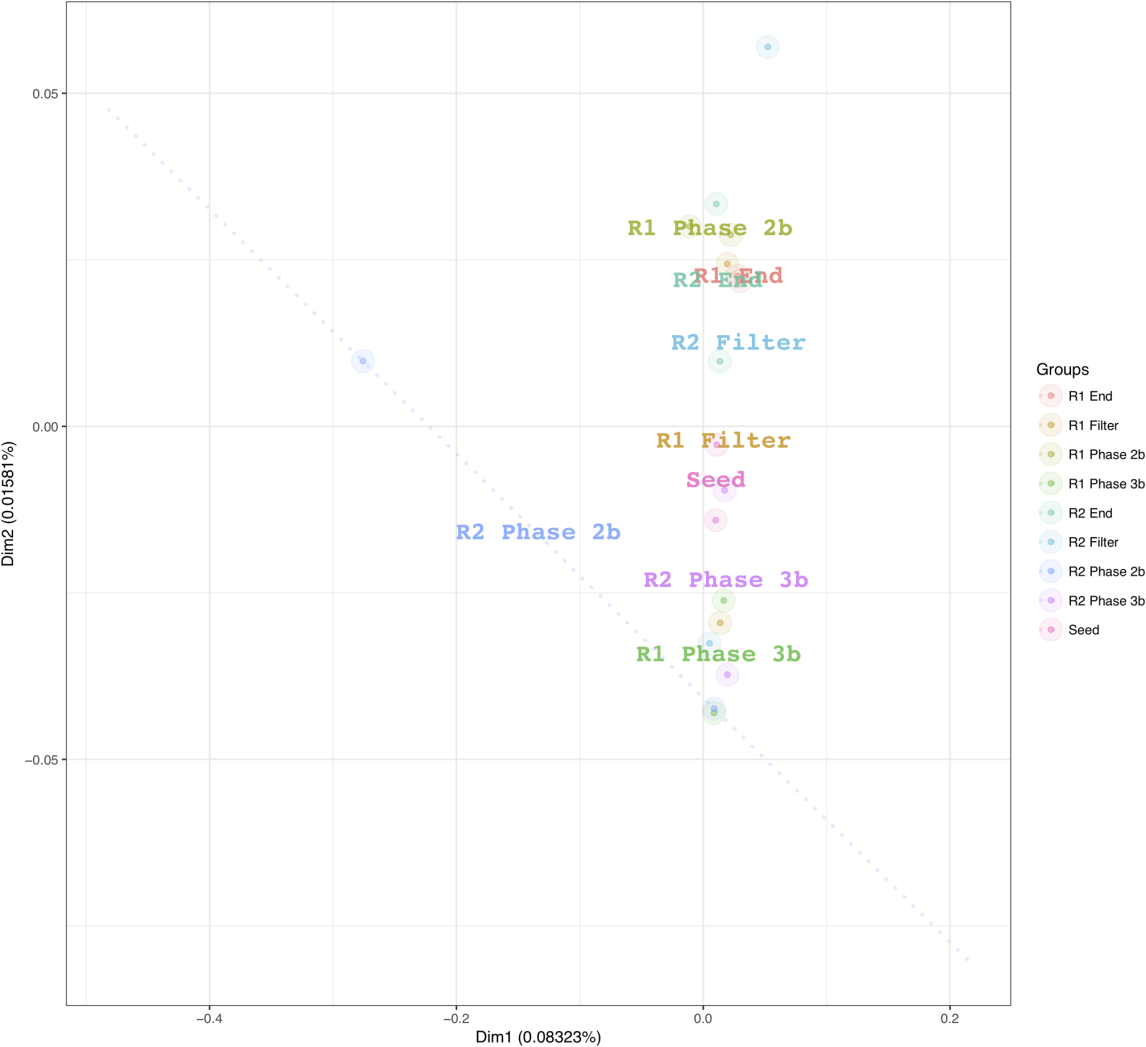
PCoA plot based on unweighted Unifrac of DNA and cDNA sequences from R1 and R2 biomass samples. For each group, the legends are drawn at the mean value of the samples of that group.

To follow the bacterial and archaeal community over time and to compare the development of the mesophilic ‘seed’ within each reactor, alpha-diversity matrices (Richness, Shannon, Simpson, Alpha and Evenness) were compared at the SEQ level. Samples from the ‘Seed’, ‘R1’ and ‘R2’ demonstrated similar observed values (Figure S1, Supporting Information). No significant difference was found between the ‘Seed’, ‘R1’ and ‘R2’; however, a large variation could be observed within the values per group. Subsequently, PCoA was carried out at SEQ level using unweighted Unifrac (ß-diversity metric) on the phylogenetic distance of sequences to visualise the similarities and dissimilarities in the microbial communities. Figure 5 demonstrates that the sequences from the replicated reactors group together based on time—‘Seed’, ‘Phase 2b’, ‘Phase 3b’, ‘Filter’ and ‘End’ rather than reactor origin. However, it must be noted that while the sequences grouped together based on sampling period they were not found to be significantly different from each other.

**Figure 6.**
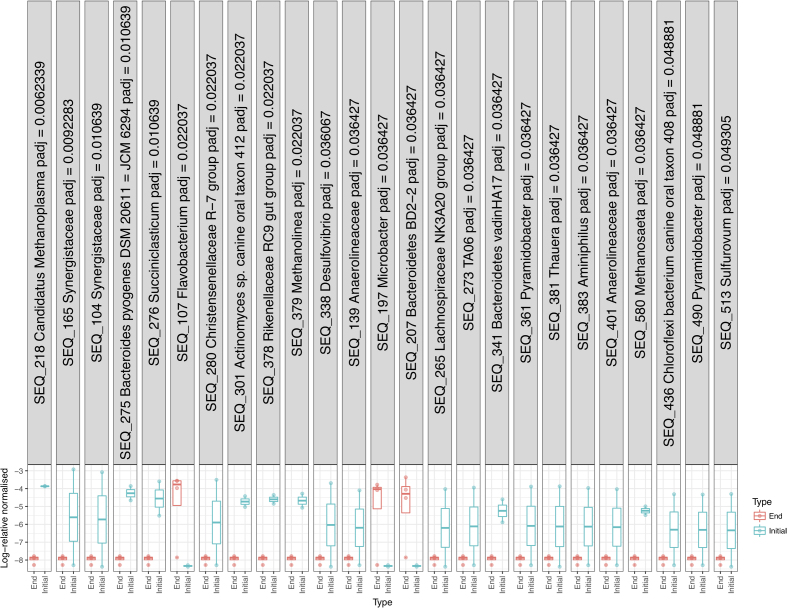
Significant SEQs contributing to beta-diversity at an SEQ level at a 1-log fold change was assessed between the seed community (Initial) and the biomass taken from the end of the trial for both R1 and R2 (End).

As the PCoA data indicated that the samples clustered based on time period, sampled analysis of significant species contributing to beta-diversity was carried out to identify what species were responsible for differences in these groupings. Analysis of the significant species contributing to beta-diversity was carried out at genus level at a 2-log and 1-log fold change for ‘Phase 2b’, ‘Phase 3b’, ‘Filter’ and ‘End’ whereby direct comparisons could be made between R1 and R2. The results demonstrated that there were no significantly different species between the replicate reactors at each of these phases (data not shown). This analysis was then repeated at SEQ level for the reactor phases (Figure S2a–c, Supporting Information). In the case of ‘Phase 2b’ SEQ4, SEQ5, SEQ6 and SEQ139 were greater in R1 (S2a, Supporting Information). SEQ4, 5 and 6 were found to be a *Methanosaeta concilii* strain X16932 and SEQ139 were found to be an uncultured *Anaerolinaceae* bacterium clone (Table S3, Supporting Information). In ‘Phase 3b’ a total of 24 SEQs were significantly different between R1 and R2 (S2b, Supporting Information). Of these 19 were greater in R1 (SEQS 36, 46, 184, 159, 17, 45, 50, 122, 210, 27, 111, 281, 19, 320, 53, 67, 103 and 92) and 5 (SEQs 69, 414, 512, 546, and 430) were greater in the R2 samples. There was no significant difference between the communities in R1 and R2 filter unit communities. There were only two sequences that were significantly different between R1 and R2 biomasses at the end of the trial (Figure S2c, Supporting Information). These were SEQ44 that was greater in R1 and SEQ235 that was greater in R2. SEQ44 was identified as an uncultured *Synergistetes* bacterium and SEQ235 was identified as *Chryseobacterium* species strain SE19. Significant species was also used to assess the maturation of the granular biofilm and the species contributing at a 1-log fold difference between the seed inoculum and the R1 and R2 end biomass (Fig. 6). From this 25 SEQS were identified as significantly different. SEQs 218, 165, 104, 275, 280, 301, 378, 379, 338, 139, 265, 273, 341, 361, 381, 383, 401, 580, 436, 490 and 513 were more abundant in the seed inoculum. While SEQs 107, 197 and 207 were more abundant in the biomass upon take down of the reactors (Fig. 6; S3, Supporting Information). Interestingly, SEQ107 was identified as a psychrotolerant species—*Flavobacterium sinopsychrotolerans* (Xu *et al.*^2011^). The identities of all significant SEQs are described in Table S3 (Supporting Information).

## DISCUSSION

Though we have not tested real sewage, we have demonstrated a sufficient capacity of the microbial community for sustained low-temperature degradation of a complex wastewater. Indeed, the removal efficiencies of these systems exceeded those reported in previous low-temperature trials carried out in a traditional UASB [upflow anaerobic sludge bed reactor] (Bandara *et al.*^2012^). This study demonstrated that a mesophilic inoculum rapidly acclimated to psychrophilic conditions to allow efficient COD removal to occur in both reactors. There were indications of a capacity for enhanced bacterial activity at 12°C, as evidenced by the protein hydrolysis assays. K*_m_* values throughout the trial increased at 12°C and decreased at 37°C, indicating an increase in substrate affinity at lower temperatures. The literature that substrate affinity will decrease at lower temperatures for psychrophiles, mesophiles and thermophiles (Nedwell 1999), but this often reflects only short-term studies. Our results point towards the emergence of psychrophilic proteolytic activity that was mirrored in both systems. While psychrophilic microorganisms may not be crucial for successful remediation of waste streams from a process steering aspect, the possibility to develop truly psychrophilic consortia could open important new opportunities for AD technology (Sekiguchi *et al.*^2001^). With respect to the archaeal populations, SMA data revealed that the microbial consortia became psychrotolerant for methanogenic substrates, rather than truly psychrophilic, a finding commonly reported in the literature (Lettinga *et al.*^1999^; O'Flaherty, Collins and Mahony 2006). Our study demonstrates that a psychrophilic or cold-adapted ‘seed’ was not necessary as a starting inoculum for successful stable anaerobic digestion at low temperatures. Bowen *et al.* (2014) reported that a mesophilic inoculum from an anaerobic suspended biomass sewage sludge reactor was not successful for LtAD, but this biomass had much lower SMA than the high-rate granular sludges used as inocula here, and in previous successful LtAD trials (e.g. Collins, Mahony and O'Flaherty 2006; Madden *et al.*^2013^; Keating *et al.*^2016^). It is likely that the retention of the anaerobic biomass in hybrid sludge bed fixed-film reactors supported the development of the reactor microbial community to function efficiently at lower temperatures. Moreover, the trial lasted 889 days, which may have provided sufficient time for the maturation of cold-adapted populations to allow for increased loading rates to be applied. This strategy for low-temperature sewage treatment offers a significant advantage over suspended biomass systems. In suspended biomass systems biomass washout would occur and the microbial population may be more sensitive to immigration and selective pressures of the influent (Vanwonterghem *et al.*^2014^).

We have demonstrated that stable, long-term, high-rate anaerobic digestion of a relatively complex wastewater, in the form of synthetic sewage, was possible and even efficient, at low-operating temperature. Reactor performance data indicated that the systems were functionally robust and stable, via the efficient effluent degradation with COD removal efficiencies for COD_Tot_ and COD_Sol_ of >73% (Table 2), despite incremental increases in the OLR applied over the course of the trial. Perhaps surprisingly, we have also shown that under these conditions hydrolysis was not rate-limiting at 12°C with evidence suggesting that COD_Sus_ in the synthetic wastewater were readily degraded to COD_Col_, despite the absence of wastewater-borne lipases associated with non-synthetic wastewaters (Petropoulos *et al.* 2017). SYNTHES was used so we could strictly define the influent and remove the variability associated with using real sewage—to be sure the microbial community development was not impacted by external variables. While SYNTHES carries a similar proportion of particulate COD (31%) to real sewage (30%) [Aiyuk and Verstraete 2004] a disadvantage is however, that starch comprises the complex carbohydrate portion, which may be easier to degrade than complex cellulosic materials that would be present in real sewage. No accumulation of solids was observed in the granular sludge bed in agreement with previous work (Keating *et al.*^2016^). The physical entrapment of solids within the pumice matrix of the hybrid reactor may have facilitated subsequent degradation.

Successful high-rate AD is contingent on well-functioning microbial communities. Stable community structures are maintained through syntrophic interactions between the bacterial and archaeal communities (Schnürer, Zellner and Svensson 1999). Low temperatures had been thought to limit these syntrophic interactions (Kotsyurbenko 2005). However, the communities represented in our systems were well balanced from the commencement of the trial, as indicated by negligible VFA accumulation in the reactor effluents and the diverse bacterial and archaeal populations found in the active fraction (cDNA-based analysis) throughout the trial. Interestingly, members of the Synergistetes were dominant members of the AD community in this trial. These are generally only found in frequencies of 1% or less in most AD systems (Godon *et al.*^2005^), but in this study their abundance increased up to 44% of bacterial sequences (Fig. 2). Isolated members of the Synergistetes partner syntrophic relationships with the methanogens in the degradation of amino acids with the production of VFAs (Vartoukian, Palmer and Wade 2007). Thus, the Synergistetes may be important for LtAD reactor function and may play a role in the low-temperature metabolism of proteins observed in reactor biomass. Perhaps, the nature of SYNTHES selected for a protein/amino acid degrading community or their high prevalence in the seed inoculum (coming from a dairy treatment facility) allowed for their development in this trial.

Adaptation involved a temporal shift in the microbial community structure over the course of the study. However, the replicate reactors maintained a remarkably similar microbial community profile to each other and this development was, in fact, reproducible down to genus level with no significant difference found between the reactors at each phase. Indeed, in the take-down biomass only two sequences were significantly different between the reactors (S3, Supporting Information). This was mirrored in the reactor performance data, whereby the reactors exhibited significant long-term reproducibility (889 days) during treatment of the synthetic sewage substrate (Table 2). Fluctuations in COD removal rates generally occurred at similar points in both reactors, indicating that degradation was occurring through biological activity, rather than by physical entrapment of the COD fractions. Two divergences in behaviour were, however, identified between the two reactors, based on process performance despite there being no significant differences between the communities at genus level. Firstly, an initial variation was observed upon start-up of the replicated systems. An immediate start-up was observed in R1 whereby all COD fractions were degraded, while the start-up of R2 took considerably longer (∼56 days). While this variation was found to be significant, no definitive cause could be identified, as molecular sampling was not carried out during the cold-adapting period so as not to disturb initial community development. Considering that COD_Sol_ removal was similar and highly efficient in both reactors during phase 1 (pointing to efficient microbial activity), the cause may have reflected a greater potential for leaching of COD_Sus_ particles or the loss of flocculent biomass from R2. Secondly, the commencement of Phase 5 led to a period of ∼5 weeks perturbation in R1, which was not mirrored in R2. qPCR data from the start of this phase showed a 1-log reduction of the total bacterial and archaeal gene copy numbers were observed in R2 (Fig. 1A). Changes in the microbial community structure were missed at this time point; however, sequencing results prior to this (from Phase 3) indicated that samples from this time point clustered together and no significant difference was found at genus level.

As stated previously there were no significant changes (1 or 2-log) in the microbial populations present between reactors at each time period at genus level as demonstrated by significant species contributing to beta-diversity analyses. This statistical measurement indicated that time was the driver of microbial community structure rather than reactor identity. Comparisons were then made at a sequence level in order to elucidate further the species that were different between the systems and the species diverging from the ‘seed’ inoculum. From the sequences outlined in Table S3 (Supporting Information), it is worth noting sequences associated with granule formation and granule integrity (*Methanosaeta concilii* species and *Anaerolinea* species). *Anaerolinea* species dominated the Chloroflexi phyla in the reactor systems. The Chloroflexi metabolise primary substrates in wastewater such as carbohydrates and cellular matter (Yamada *et al.*^2005^). *Anaerolinea* species belong to Subphylum 1 an elusive phylum comprising environmental clones (Hugenholtz, Goebel and Pace 1998). They form web-like structures on the outside of granules in mesophilic and thermophilic systems and thus are thought to be important for granule structure (Sekiguchi *et al.*^1998^). Given their stable dominance in these reactors further characterisation of their role in low-temperature systems would be valuable. *Methanosaeta* dominated the archaeal communities in both systems as demonstrated by sequencing analysis (Figs 2 and 4; Table S3, Supporting Information). *Methanosaeta concilii* is a key organism in granulation in these anaerobic systems (Hulshoff Pol *et al.*^2004^). The distinctive solely acetate utilising acetoclastic *Methanosaeta* are known to dominate in steady state reactors in which acetate concentrations are low (McMahon *et al.*^2001^). VFA analyses indicated that in-reactor acetate values were negligible throughout the trial. Moreover, acetoclastic methanogens have been seen to be dominant at low temperatures (Chin, Lukow and Conrad 1999). However, this is in contrast to reports by several authors that suggest that acetoclastic activity is impacted by lower temperatures and that under these conditions hydrogenotrophic methanogens dominate and facilitate efficient VFA degradation (Nozhevnikova *et al.*^2000^; Collins *et al.*^2005^; Connaughton, Collins and O'Flaherty 2006). In Phase 3, *Anaerolinea*-like species were reduced in R1 in comparison to R2 (following this R1 demonstrated biomass washout upon commencement of Phase 5). Biomass sampled from Phase 2 demonstrated that *Methanosaeta* like species were reduced in R2 in comparison to R1 (prior to this phase R2 demonstrated reduced performance). While this data are not conclusive, the close monitoring of such species is crucial to granule integrity may provide an opportunity to link granule health with process performance and to develop means to promote their growth in poorly performing systems.

## CONCLUSIONS

Overall this study revealed that a cold-adapted or psychrophilic ‘seed’ inoculum was not necessary for efficient LtAD of wastewater. Our work demonstrated reproducible process performance and mirrored microbial community development between replicated systems. The nature of the reactor system allowed for the retention of biomass allowing sufficient cold-adapted communities to develop and mature, to the point where increased activity at low temperature developed within the hydrolytic and methanogenic populations. Next generation sequencing identified a number of possible cold-adapted species and increased abundance of the *Synergistetes* and *Anaerolinea* phyla that warrant further targeted investigations to determine their possible future biotechnological relevance.

## Supplementary Material

Supplementary Data
