# Supplementary material for: Cold adaptation and replicable microbial community development during long-term low-temperature anaerobic digestion treatment of synthetic sewage
Source: FEMS Microbiol Ecol. 2018 May 25;94(7):fiy095. doi: 10.1093/femsec/fiy095 (PMC5995215; doi:10.1093/femsec/fiy095)
Supplement: Supplementary Data [file fiy095_supplemental_files.zip › Supplementary Information S3.docx]

**S3.** SEQS identified as significant through contribution to beta-diversity analyses. SEQS were blasted against NCBI database and compared to cultured and environmental/uncultured isolates.

| **Sample** | **SEQ no.** | **Query Cover** | **Identity Cover** | **Accession no.** | **Description** |
| --- | --- | --- | --- | --- | --- |
| **Phase 2b**  **>R1** | **4** | 95% | 99% | KM408635.1 | *Methanosaeta concilii* strain X16932 16S ribosomal RNA gene, partial sequence |
|  | **5** | 95% | 99% | KM408635.1 | Same as above |
|  | **6** | 95% | 99% | KM408635.1 | Same as above |
|  | **139** | 95% | 97% | DQ168648.1 | Bacterium JN18_A7_F* 16S ribosomal RNA gene, partial sequence |
| **Phase 3b** |  |  |  |  |  |
| **> R1** | **36** | 100% | 86% | NR_145867.1 | *Hypnocyclicus thermotrophus* strain IR-2 16S ribosomal RNA, partial sequence |
|  |  | 100% | 100% | JN387540.1 | Uncultured microorganism clone Group62_g 16S ribosomal RNA gene, partial sequence |
|  | **46** | 100% | 90% | HQ663240.1 | Bacteroidetes bacterium SCGC AAA028-D13 16S small subunit ribosomal RNA gene, partial sequence |
|  | **184** | 100% | 97% | HF559181.1 | *Geothrix fermentans* partial 16S rRNA gene, strain HradG1, clone 8 |
|  | **159** | 98% | 93% | KX261405.1 | *Leptolinea tardivitalis* isolate HIT2 16S ribosomal RNA gene, partial sequence |
|  | **17** | 100% | 99% | NR_026336.1 | *Clostridium vincentii* strain DSM 10228 16S ribosomal RNA gene, partial sequence |
|  | **45** | 100% | 94% | CP012159.1 | *Chondromyces crocatus* strain Cm c5, complete genome |
|  |  | 100% | 100% | LN650985.2 | Uncultured bacterium partial 16S rRNA gene, clone Clone_2 |
|  | **50** | 100% | 97% | Y09280.1 | *T.baregensis* 16S rRNA gene |
|  | **122** | 100% | 99% | AB910740.1 | *Paludibacter propionicigenes* gene for 16S ribosomal RNA, partial sequence |
|  |  | 100% | 100% | KT072573.1 | Uncultured bacterium clone SLV_3WBAC_93 16S ribosomal RNA gene, partial sequence |
|  | **210** | 100% | 98% | HQ290497.1 | Bacterium SCGC AAA018-I2 small subunit ribosomal RNA gene, partial sequence |
|  |  | 100% | 100% | LT625320.1 | Uncultured *Methylococcaceae* bacterium partial 16S rRNA gene, isolate OTU 421 |
|  | **27** | 100% | 100% | KY818302.1 | *Acinetobacter* sp. strain RPSL-2 16S ribosomal RNA gene, partial sequence |
|  | **111** | 100% | 100% | KX585901.1 | *Aeromonas veronii* strain QD160502 16S ribosomal RNA gene, partial sequence |
|  | **281** | 100% | 100% | KY438547.1 | *Lactococcus* sp. strain AP363 16S ribosomal RNA gene, partial sequence |
|  | **19** | 100% | 100% | LC306852.1 | *Carnobacterium maltaromaticum* gene for 16S ribosomal RNA, partial sequence, strain: JCM 9135 |
|  | **320** | 100% | 100% | KX129799.1 | *Methylomonas* sp. EbB 16S ribosomal RNA gene, partial sequence |
|  | **53** | 100% | 99% | NR_074975.1 | *Pelobacter propionicus* strain DSM 2379 16S ribosomal RNA gene, complete sequence |
|  |  | 100% | 100% | LK024824.2 | Uncultured bacterium partial 16S rRNA gene, clone BEtH4P6D04 |
|  | **67** | 100% | 93% | LC271159.1 | *Clostridium* sp. AY5-bB4 gene for 16S ribosomal RNA, partial sequence |
|  |  | 100% | 100% | FN396935.1 | Uncultured bacterium partial 16S rRNA gene, clone 8A |
|  | **103** | 100% | 89% | HQ663240.1 | Bacteroidetes bacterium SCGC AAA028-D13 16S small subunit ribosomal RNA gene, partial sequence |
|  | **92** | 100% | 91% | KT619174.1 | *Azospirillum* sp. EP3-3L 16S ribosomal RNA gene, partial sequence |
| **> R2** | **69** | 100% | 95% | NR_040971.1 | *Leptolinea tardivitalis* strain YMTK-2 16S ribosomal RNA gene, partial sequence |
|  | **414** | 100% | 91% | KP174524.1 | Bacterium YC-ZSS-LKJ180 16S ribosomal RNA gene, partial sequence |
|  | **512** | 100% | 91% | KP174642.1 | Bacterium YC-LK-LKJ31 16S ribosomal RNA gene, partial sequence |
|  |  | 100% | 100% | KF990102.1 | Uncultured bacterium clone BS79 16S ribosomal RNA gene, partial sequence |
|  | **546** | 100% | 100% | LC049960.1 | Bacteroidales bacterium TBC1 gene for 16S ribosomal RNA, partial sequence |
|  | **430** | 100% | 92% |  | *Anaerolineaceae* bacterium CAMBI-1 genome assembly, chromosome: I |
|  |  | 100% | 100% | AB291288.1 | Uncultured bacterium gene for 16S rRNA, partial sequence, clone: SwB2 |
| **End** |  |  |  |  |  |
| **> R1** | **235** | 95% | 99% | KY476631.1 | *Chryseobacterium* sp. strain SE19 16S ribosomal RNA gene, partial sequence |
| **> R2** | **44** | 95% | 98% | KF528158.1 | *Selenomonas sputigena* strain 4A 16S ribosomal RNA gene, partial sequence |
| **End vs Initial** |  |  |  |  |  |
| **> Initial** | **218** | 95% | 99% | JQ267998.1 | Archaeon PY-4 16S ribosomal RNA gene, partial sequence |
|  | **165** | 100% | 98% | KF528158.1 | Selenomonas sputigena strain 4A 16S ribosomal RNA gene, partial sequence |
|  | **104** | 100% | 98% | KF528158.1 | *Selenomonas sputigena* strain 4A 16S ribosomal RNA gene, partial sequence |
|  |  | 100% | 100% | LT841469.1 | Uncultured Synergistaceae bacterium partial 16S rRNA gene, isolate BACT_OTU_45 |
|  | **275** | 95% | 99% | HF558365.1 | *Bacteroides pyogenes* partial 16S rRNA gene, strain B38024 |
|  | **280** | 95% | 94% | U81761.2 | Unidentified eubacterium clone vadinBB35 16S ribosomal RNA gene, partial sequence |
|  | **301** | 100% | 99% | LT671587.1 | *Actinomyces* sp. Marseille-P3561 partial 16S rRNA gene, strain Marseille-P3561 |
|  | **378** | 95% | 91% | CP011998.1 | *Ralstonia solanacearum* strain YC45 plasmid, complete sequence |
|  | **379** | 100% | 98% | KP109880.1 | *Methanolinea tarda* clone BC-13 16S ribosomal RNA gene, partial sequence |
|  |  | 100% | 100% | KT167062.1 | Uncultured archaeon clone WRParc5 16S ribosomal RNA gene, partial sequence |
|  | **338** | 100% | 100% | AM946979.1 | *Desulfovibrio* sp. S14 PV-2008 partial 16S rRNA gene, strain S14 |
|  | **139** | 95% | 97% | DQ168648.1 | Bacterium JN18_A7_F* 16S ribosomal RNA gene, partial sequence |
|  | **265** | 95% | 99% | KF698340.1 | Bacterium V9D2006 16S ribosomal RNA gene, partial sequence |
|  | **273** | 100% | 82% | KY363637.1 | *Conexibacter* sp. strain Seoho-28 16S ribosomal RNA gene, partial sequence |
|  | **341** | 95% | 90% | KP174642.1 | Bacterium YC-LK-LKJ31 16S ribosomal RNA gene, partial sequence |
|  | **361** | 95% | 99% | MF361106.1 | *Pyramidobacter* sp. strain CG50-5 16S ribosomal RNA gene, partial sequence |
|  | **381** | 95% | 99% | EU434525.1 | *Thauera phenylacetica* strain b208 16S ribosomal RNA gene, partial sequence |
|  | **383** | 100% | 100% | KJ638711.1 | *Synergistaceae* bacterium JGI 0000059-A07 16S ribosomal RNA gene, partial sequence |
|  | **401** | 95% | 97% | DQ168648.1 | Bacterium JN18_A7_F* 16S ribosomal RNA gene, partial sequence |
|  | **580** | 96% | 99% | NR_074177.1 | *Methanospirillum hungatei* strain JF-1 16S ribosomal RNA gene, complete sequence |
|  | **436** | 95% | 99% | KM018331.1 | *Chloroflexi* bacterium canine oral taxon COT-408 clone 5U46 16S ribosomal RNA gene, partial sequence |
|  | **490** | 95% | 97% | MF361106.1 | *Pyramidobacter* sp. strain CG50-5 16S ribosomal RNA gene, partial sequence |
|  | **513** | 95% | 98% | AJ431218.1 | Proteobacterium Dex60-82 16S rRNA gene, strain Dex60-82 |
| **> End** | **107** | 95% | 99% | KX809750.1 | *Flavobacterium sinopsychrotolerans* strain KS1-31 16S ribosomal RNA gene, partial sequence |
|  | **197** | 95% | 94% | LC056021.1 | *Porphyromonadaceae* bacterium H1 gene for 16S ribosomal RNA, partial sequence |
|  | **207** | 95% | 90% | KX417406.1 | *Marinilabilia* sp. FdP-HMN_10-2-6 16S ribosomal RNA gene, partial sequence |
| **Archaeal** | **1** | 100% | 100% | KM408635.1 | *Methanosaeta concilii* strain X16932 16S ribosomal RNA gene, partial sequence |
| **heatmap** | **3, 30 and 510** | 100% | 100% | AY552778.3 | *Methanobacterium beijingense* strain 4-1 16S ribosomal RNA gene, partial sequence |
| **SEQS** | **4, 5, 6 and 186** | 95% | 99% | KM408635.1 | *Methanosaeta concilii* strain X16932 16S ribosomal RNA gene, partial sequence |
|  | **7** | 100% | 100% | KP109878.1 | *Methanobacterium beijingense* clone BC-7 16S ribosomal RNA gene, partial sequence |
|  | **8 and 29** | 100% | 100% | MF992205.1 | *Methanobacterium subterraneum* strain GO3 16S ribosomal RNA gene, partial sequence |
|  | **23 and 296** | 100% | 100% | KX344121.1 | *Methanobacterium formicicum* strain L21-2 16S ribosomal RNA gene, partial sequence |
|  | **90, 114, 379 and 579** | 96% | 98% | KP109880.1 | *Methanolinea tarda* clone BC-13 16S ribosomal RNA gene, partial sequence |
|  | **106** | 95% | 99% | LT625971.1 | Uncultured *Methanomassiliicoccus* sp. partial 16S rRNA gene, isolate OTU 207 |
|  | **218** | 95% | 99% | LT624813.1 | Uncultured *Thermoplasmatales* archaeon partial 16S rRNA gene, isolate OTU_9 |
|  | **385 and 611** | 96% | 99% | NR_133782.1 | *Methanospirillum psychrodurum* strain X-18 16S ribosomal RNA, partial sequence |
