# Supplementary figures and images for: Cold adaptation and replicable microbial community development during long-term low-temperature anaerobic digestion treatment of synthetic sewage
Source: FEMS Microbiol Ecol. 2018 May 25;94(7):fiy095. doi: 10.1093/femsec/fiy095 (PMC5995215; doi:10.1093/femsec/fiy095)

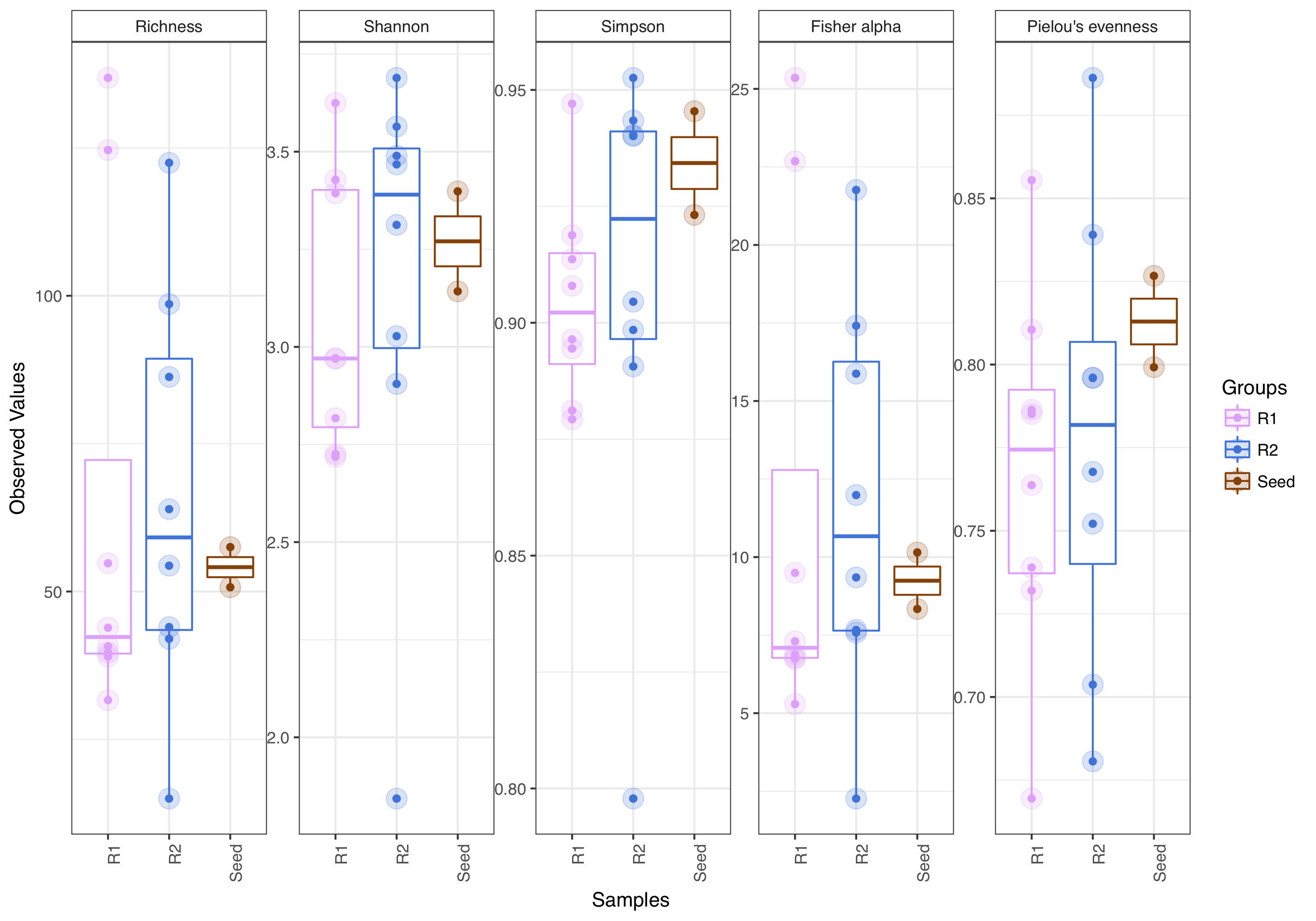

Supplement: Supplementary Data [file fiy095_supplemental_files.zip › Supplementary Information S1.tiff]

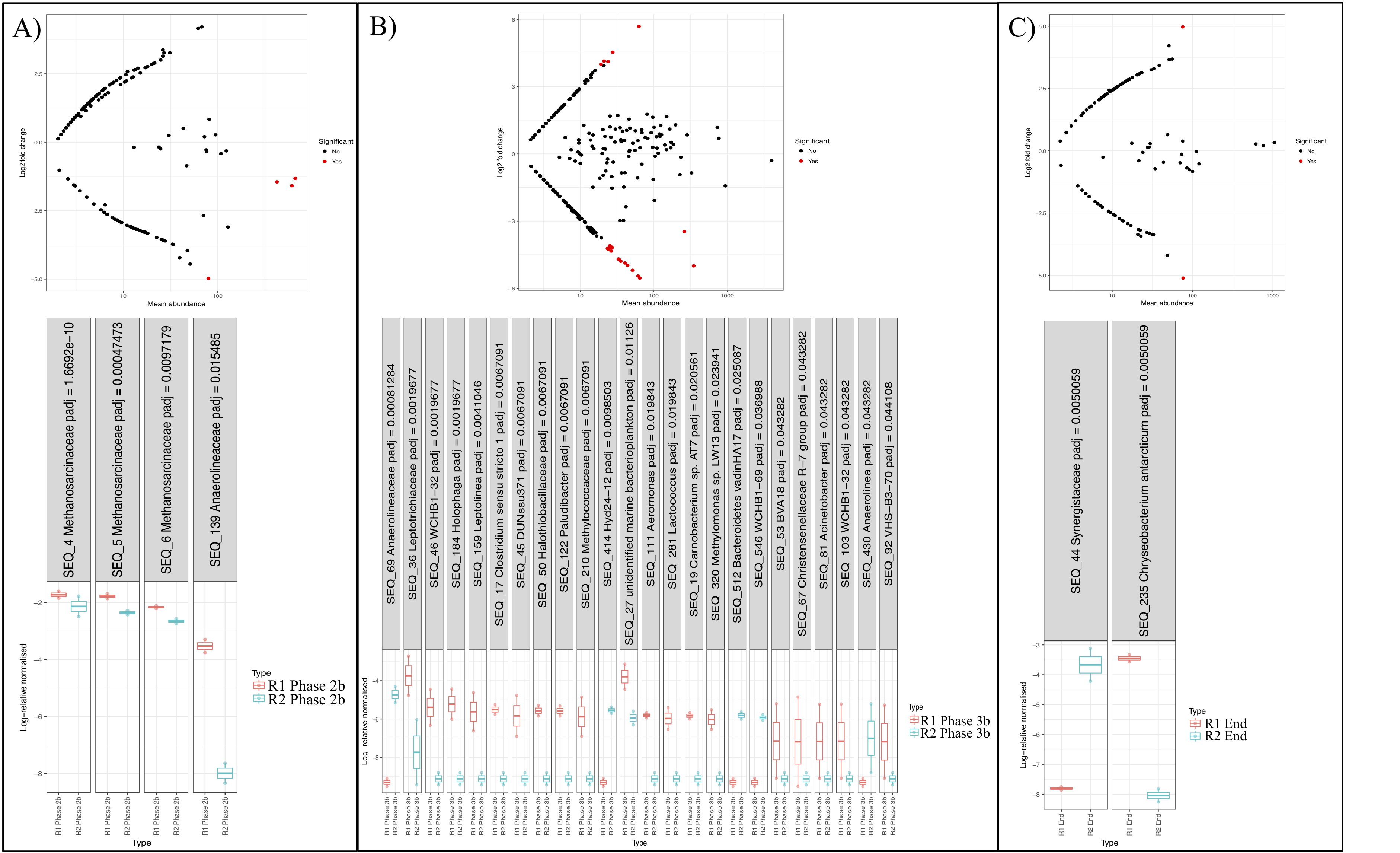

Supplement: Supplementary Data [file fiy095_supplemental_files.zip › Supplementary Information S2.tiff]
